# Role of T-2 toxin in the modulation of oxidative homeostasis and immune function in three-dimensional hepatic cell cultures of chicken origin

**DOI:** 10.3389/fvets.2026.1759841

**Published:** 2026-02-10

**Authors:** Júlia Vörösházi, Máté Mackei, Csilla Sebők, Patrik Tráj, Rege A. Márton, Zsuzsanna Neogrády, Gábor Mátis

**Affiliations:** 1 Division of Biochemistry, Department of Physiology and Biochemistry, University of Veterinary Medicine, Budapest, Hungary; 2 National Laboratory of Infectious Animal Diseases, Antimicrobial Resistance, Veterinary Public Health and Food Chain Safety, University of Veterinary Medicine, Budapest, Hungary

**Keywords:** 3D cell cultures, *in vitro*, mycotoxin, poultry, T-2 toxin, trichothecene

## Abstract

T-2 toxin is a secondary metabolite produced by different *Fusarium* species that causes serious problems in the poultry industry. It can damage several organ systems, mainly by inhibiting protein synthesis, and is also likely to induce oxidative stress in cells. In this study, the adverse effects of T-2 toxin were investigated in magnetic three-dimensional hepatic cell cultures of chicken origin. Cultures were treated with 100, 500, and 1,000 nM T-2 toxin for 48 and 72 h. The metabolic activity of the cells was determined using CCK-8 assay. To investigate the changes in the oxidative homeostasis, the levels of MDA, HSP27, and 8-OHdG were measured. To monitor immune response, the concentrations of IL-6 and 8 were determined. The metabolic activity of the cells decreased after the toxin treatments, but this decrease was alleviated after 72 h. Both MDA and HSP27 levels were significantly diminished by T-2 toxin after 48 h. The concentration of 8-OHdG was reduced by 72 h exposure to 100 nM T-2 toxin. In the case of IL-6, the 1,000 nM T-2 toxin was able to decrease its levels after 48 and 72 h, whereas IL-8 levels were lowered after 72 h by all three toxin treatments. Overall, these results suggest that T-2 toxin had a negative effect on the cell function of the hepatic spheroids, but it was not cytotoxic. The cells showed metabolic adaptation to the 72 h treatment and possessed effective compensation against oxidative stress while eliciting an immunosuppressive effect on the liver cells. These findings indicate that subclinical T-2 toxin exposure may impair liver metabolism and the immune system in poultry, highlighting the importance of regular mycotoxin monitoring and elimination techniques from the feed.

## 1 Introduction

Fungi species generate a variety of secondary metabolites, including mycotoxins, which pose significant toxicological risks to both animals and humans (1). An important group among these is the trichothecene mycotoxins produced by different *Fusarium* species (2). These fungi are predominantly found in cooler, humid climatic regions and are frequently associated with infections of cereal crops, such as maize, wheat, and oat (3). Within the trichothecenes, T-2 toxin is considered one of the most toxic contaminants affecting various crops worldwide (4). Due to its resistance to both heat and chemical treatments, preventing contamination is challenging. Nevertheless, by understanding its exact molecular mechanism of action, the degree of toxicity can be reduced (5).

The mostly cereal-consuming poultry species may come into regular contact with T-2 toxin (6). Although avian species can rapidly metabolize T-2 toxin, making them less susceptible to its harmful effects than mammals, both acute and chronic exposure can still have detrimental effects on their health (7, 8). The toxin has been shown to adversely affect the performance and immune competence of broilers, even at subclinical exposure levels (9). The main effects of T-2 toxin in poultry include reduced feed intake, impaired weight gain and nutritional efficiency, immune modulation, as well as neurological and reproductive disorders (10, 11). These performance losses are closely linked to immunosuppressive effects, as T-2 toxin can induce lymphoid organ atrophy, suppress antibody production, and alter leukocyte populations, leading to increased susceptibility to infectious diseases (12–14). Previous studies have demonstrated that T-2 toxin exposure reduces both humoral and cell-mediated immune responses in broilers, including reduced vaccine responses and altered lymphocyte function (15, 16).

The primary toxic mechanism of T-2 toxin is cytotoxicity, which contributes to immunotoxicity, metabolic disruption, and hepatotoxicity (17). At the cellular level, T-2 toxin inhibits protein synthesis by binding the peptidyl transferase enzyme of the 60S ribosomal subunit, thereby blocking transpeptidation and peptide-bond formation. This means the disruption of the elongation and termination stages of protein synthesis (8, 18). Furthermore, T-2 toxin inhibits DNA and RNA synthesis, affects the cell cycle, and ultimately induces apoptosis (8, 19, 20). In addition, it increases the production of reactive oxygen species (ROS), leading to oxidative stress and the induction of mitochondrial-mediated apoptosic pathways (20, 21). The development of oxidative stress also alters the cellular levels of oxidative parameters, such as malondialdehyde (MDA), a product of the peroxidation of membrane lipids (17, 22).

Another oxidative parameter is the heat shock protein 27 (HSP27), an ATP-independent chaperone and a member of the small heat shock protein (sHSP) family (23, 24). This protein binds to misfolded or oxidized polypeptides and creates a reservoir for them, inhibits aggregation, and promotes the refolding of denatured proteins (24, 25). HSP27 also stimulates the production of several enzymes involved in the maintenance of redox homeostasis, including glucose-6-phosphate dehydrogenase or glutathione S-transferase. Through these mechanisms, HSP27 exerts a protective, ROS-reducing effect against oxidative stress (25).

ROS production stimulated by T-2 toxin can also cause oxidative damage to the DNA, a relevant indicator of which could be 8-hydroxy-2′-deoxyguanosine (8-OHdG) (26). In the process, the generated hydroxyl radicals attack the DNA strands, producing C8-hydroxyguanine (8-OHGua) or its nucleoside form, 8-OHdG (26). 8-OHdG often undergoes keto-enol tautomerism to become 8-oxo-7,8-dihydro-2′-deoxyguanosine (8-oxodG) (26). DNA oxidation can lead to several deleterious consequences, including the loss of base pairing specificity of 8-OHdG and the development of mutations (26).

Another main target of T-2 toxin is the immune system, which is influenced in a dose- and time-dependent manner (27). Inflammatory cytokines play a central role in the mechanism of action of the toxin, and their production is affected by the toxin concentration and the time of exposure (28, 29). Previous studies have reported elevated levels of interleukin- (IL-) 1*α*, IL-1*β*, and IL-6, as well as tumour necrosis factor-α (TNF-α) in the brain and spleen of mice exposed to T-2 toxin (30).

Similarly, increased expression of IL-1α, IL-β, IL-6, IL-11, and TNF-α has been observed in rat liver cell line; this effect, combined with altered DNA methylation, significantly enhanced the hepatotoxicity of T-2 toxin (29). Furthermore, in porcine models, the toxin stimulated the expression of IL-2, IL-12, IL-17A, and interferon-*γ* (IFN-γ), while also inducing anti-inflammatory cytokines, such as IL-4 and IL-10 (31). In contrast, it reduced the production of some cytokines, for instance, that of IL-1β, IL-4, IL-10, IL-12, and TNF-α, as demonstrated *in vitro* in mice (32). All these seemingly contradictory results underline the importance of further investigating the immunomodulatory effects of T-2 toxin.

Traditional two-dimensional (2D) cell models have long been used in *in vitro* research to study hepatotoxicity. However, the effectiveness of 2D primary hepatic cell cultures is limited because of the rapid dedifferentiation of the cells and the loss of hepatocyte-specific functions. In addition, their short-term viability restricts their use mainly to acute exposure studies (33). Three-dimensional (3D) cell models are nowadays becoming widely used in *in vitro* studies because they better mimic the *in vivo* conditions and can be maintained for up to several weeks (34). Their organ-like sturcutre and morphology, as well as physiological relevance make them a suitable model for drug response and toxicity research. Increasing evidence also indicates that 3D models respond differently to drugs or stimuli than traditional 2D models because of the involvement of different metabolic processes (35, 36).

In this study, magnetic 3D bioprinting was used to create primary 3D hepatic cell co-cultures of chicken origin. This technique involves the use of nanoparticles consisting of iron oxide, gold, and poly-L-lysine to magnetize the cells. The poly-L-lysine of the nanoparticles binds to the cell membranes, and upon exposure to an external magnetic field, the magnetized cells aggregate into spheroids at the bottom of a multiwell plate (37).

This research aimed to demonstrate that cell cultures created using magnetic 3D bioprinting can be maintained longer than traditional cell cultures without affecting the viability of the cells. The effects of the abovementioned prolonged T-2 toxin exposure on oxidative stress, further on immune and inflammatory processes were also intended to be investigated in the established chicken-derived *in vitro* model.

## 2 Materials and methods

### 2.1 Animal welfare statement

The authors confirm that they are following the journal’s ethical rules, as stated on the author guidelines page, and that they received the necessary permission from the ethical review committee. The authors declare that they were in line with the EU guidelines for the protection of animals used in this research. The work with animals was confirmed by the Government Office of Zala County, Food Chain Safety, Plant Protection and Soil Conservation Directorate, Budapest, Hungary (permission number: ZAI/040/00522–7/2020, date: 5/11/2020).

### 2.2 Isolation of the cells

All reagents used in the present study were obtained from Merck KGaA (Darmstadt, Germany) unless otherwise specified. Following a thorough veterinary evaluation, the chickens were found to be in good health and suitable for the isolation of hepatic cells. Cells were isolated from three-week-old male Ross 308 broiler chicken (obtained from Gallus Poultry Farming and Hatching Ltd., Devecser, Hungary) following the protocol developed by our research group (9, 38, 39). The animal was anaesthetized with CO_2_, and a three-step perfusion of the liver was carried out via the *vena gastropancreaticoduodenalis*. First, the liver was flushed with 150 mL of 0.5 nM ethylene glycol tetraacetic acid (EGTA)-containing Hanks’ Balanced Salt Solution (HBSS), followed by 150 mL of EGTA-free HBSS. To degrade the extracellular matrix and disrupt the organ for the release of the liver cells, 130 mL of MgCl_2_ and CaCl_2_ (both 7 mM) HBSS supplemented with 1 mg/mL type IV collagenase (Nordmark, Uetersen, Germany) was also rinsed through the liver.

Following the disruption of the Glisson’s capsule, the liver cells were resuspended in 50 mL ice-cold bovine serum albumin (BSA, 2.5%)-containing HBSS. The remaining cell aggregates and the undigested interstitium were removed by filtering the gained suspension through 3 layers of sterile gauze. Hepatocytes and non-parenchymal (NP) cells (mostly macrophages) were separated using differential centrifugation, resuspended in William’s Medium E.

Cell viability and total cell number were determined in a Bürker chamber using trypan blue staining. Viability exceeded 90% for both cell types. The hepatocyte-NP cell mixture was then set to a final concentration of 5 × 10^5^ cells/ml, and the hepatocyte to NP cell ratio was adjusted to 6:1.

Reagents and equipment for the magnetic 3D bioprinting were purchased from Greiner Bio-One Hungary Ltd. (Mosonmagyaróvár, Hungary). The preparation of the 3D cell cultures followed the protocol established by our research group (9, 39). To magnetize the cells, 800 μL of magnetic nanoparticles (NanoShuttle™-PL) were added to 8 mL of hepatocyte-NP cell mixed suspension. The cells were then seeded onto a 96-well cell-repellent plate and incubated for 1 h at 37 °C, allowing the nanoparticles to bind to the cell membranes. Subsequently, the plate was placed on top of a so-called Spheroid Drive and incubated for 48 h at 37 °C in a humid atmosphere with 5% CO_2_. After 24 h, the culture media was replaced with serum-free medium using a Holding Drive.

### 2.3 Study design

The study design was based on a previous experiment with 24 h of T-2 toxin (cat.no.: T4887-25MG) treatment performed by our research group (9). Following the 48 h incubation, the cells were exposed to culture media supplemented with 0 (control), 100, 500, and 1,000 nM T-2 toxin for 48 and 72 h. Cell culture media samples were collected at the end of the incubations, and cells were lysed after 72 h by intermittent sonication (1/s) in 40 μL of M-PER buffer for 5 s using a Bandelin Sonopuls HD 2200 homogenizer (Bandelin Electronic GmbH & Co. KG, Berlin, Germany). Samples were kept at −80 °C until further analysis.

### 2.4 Measurements

#### 2.4.1 Metabolic activity of the cells

The metabolic activity of the cells was assessed using Cell Counting Kit-8 (CCK-8) assay (Dojindo Molecular Technologies, Rockville, MD, USA) according to the manufacturer’s instructions. This assay detects the amount of reduced coenzymes produced during the cellular catabolic processes. 10 μL of CCK-8 reagent was added to 100 μL of the cell culture media in each well of a 96-well plate after both 48 and 72 h incubation. After a 4 h incubation with the CCK-8 reagent, the absorbance at 450 nm was measured using a Multiskan GO 3.2 reader (Thermo Fisher Scientific, Waltham, MA, USA).

#### 2.4.2 Cellular inflammation, oxidative stress, and DNA damage

All further measurements were conducted using commercially available chicken-specific ELISA kits (MyBioSource, San Diego, CA, USA) according to the manufacturer’s protocols. To investigate the oxidative stress, the concentrations of MDA and HSP27 were measured from the cell culture media. To assess potential DNA damage resulting from oxidative stress, the amount of 8-OHdG was assayed from the cell culture lysates after 72 h of treatment. Additionally, the effects of T-2 toxin on cellular inflammation were determined by measuring the concentrations of two pro-inflammatory cytokines, IL-6 and IL-8 from the cell culture media.

### 2.5 Statistical analysis of data

Statistical analysis was performed using the R 4.3.1 software (R Core Team 2023). Each treatment group contained 15 replicates (n = 15/group). Shapiro–Wilk test and Levene’s test were applied to verify normal distribution and homogeneity of variance, respectively. Group differences were assessed using one-way analysis of variance (ANOVA) and Dunett’s *post hoc* tests for pairwise comparisons. Treatment groups were compared to the control group, and results were expressed as mean ± standard error of the mean (SEM). Differences were considered significant at *p < 0.05*. Results were visualized using GraphPad Prism version 9.1.2. for Windows (GraphPad Software, San Diego, CA, USA). Results were visualized with relative values. For the group means, see Supplementary Table 1.

## 3 Results

### 3.1 Metabolic activity

**Figure 1:**
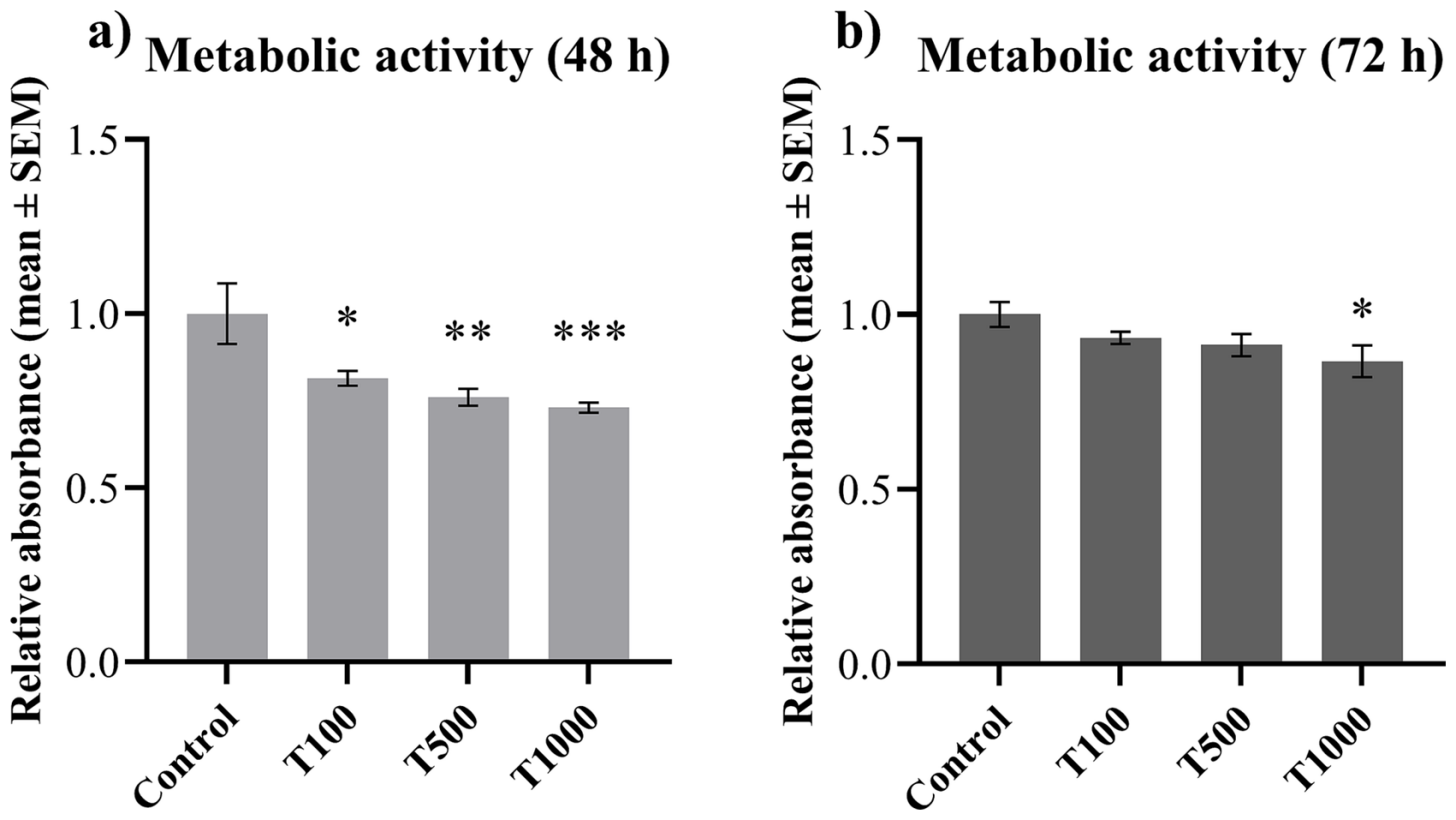
Effects of 48 and 72 h T-2 toxin treatments on the metabolic activity of primary hepatic 3D cell co-cultures of chicken origin, assessed by CCK-8 test. Cellular metabolic activity after **(a)** 48 h and **(b)** 72 h of treatment. Control: cells without T-2 toxin exposure; T100: 100 nM, T500: 500 nM, T1000: 1000 nM T-2 toxin treatments. Relative absorbances were calculated by considering the mean value of the control group as 1. Results are expressed as mean ± SEM. Significant difference from the control group is indicated by *. * *p* < 0.05; ** *p* < 0.01; *** *p* < 0.001.

Metabolic activity of the cultured cells was measured by CCK-8 test. No significant changes were observed between the control groups of the different incubation periods (0.194 ± 0.017 and 0.171 ± 0.006 for 48 and 72 h control groups, respectively, with *p* = 0.163). After 48 h, all three T-2 toxin treatments significantly reduced (*p* = 0.017, *p* = 0.004, *p* < 0.001, respectively) the metabolic activity of the cells (Figure 1a). After the 72 h incubation, only the highest T-2 toxin treatment decreased significantly (*p* = 0.035) the cellular metabolic activity (Figure 1b). The mean ± SEM values obtained from the CCK-8 measurement and the corresponding significances are shown in Supplementary Table 1.

### 3.2 Oxidative stress

**Figure 2:**
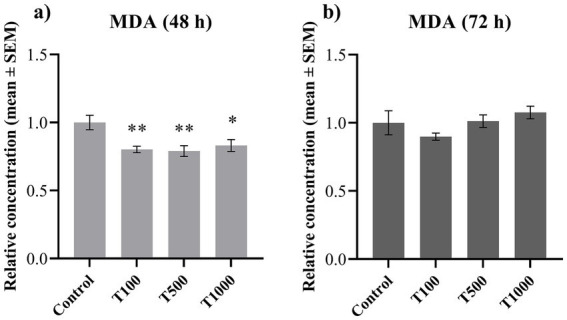
Effects of 48 and 72 h T-2 toxin treatments on the malondialdehyde (MDA) content of primary hepatic 3D cell co-cultures of chicken origin, assessed by chicken-specific ELISA tests. Concentration of MDA after **(a)** 48 h and **(b)** 72 h of treatment. Control: cells without T-2 toxin exposure; T100: 100 nM, T500: 500 nM, T1000: 1000 nM T-2 toxin treatments. Relative concentrations were calculated by considering the mean value of the control group as 1. Results are expressed as mean ± SEM. Significant difference from the control group is indicated by *. * *p* < 0.05; ** *p* < 0.01.

**Figure 3:**
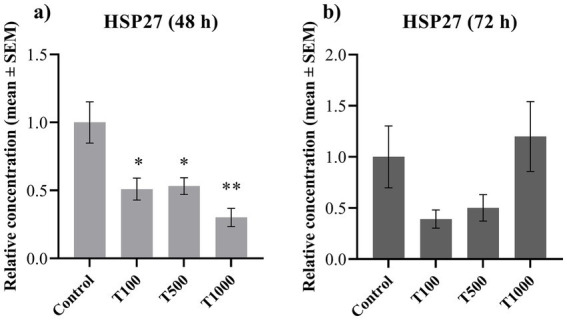
Effects of 48 and 72 h T-2 toxin treatments on the heat shock protein 27 (HSP27) content of primary hepatic 3D cell co-cultures of chicken origin, assessed by chicken-specific ELISA tests. Concentration of HSP27 after **(a)** 48 h and **(b)** 72 h of treatment. Control: cells without T-2 toxin exposure; T100: 100 nM, T500: 500 nM, T1000: 1000 nM T-2 toxin treatment. Relative concentrations were calculated by considering the mean value of the control group as 1. Results are expressed as mean ± SEM. Significant difference from the control group is indicated by *. *p < 0.05; ** *p* < 0.01.

**Figure 4:**
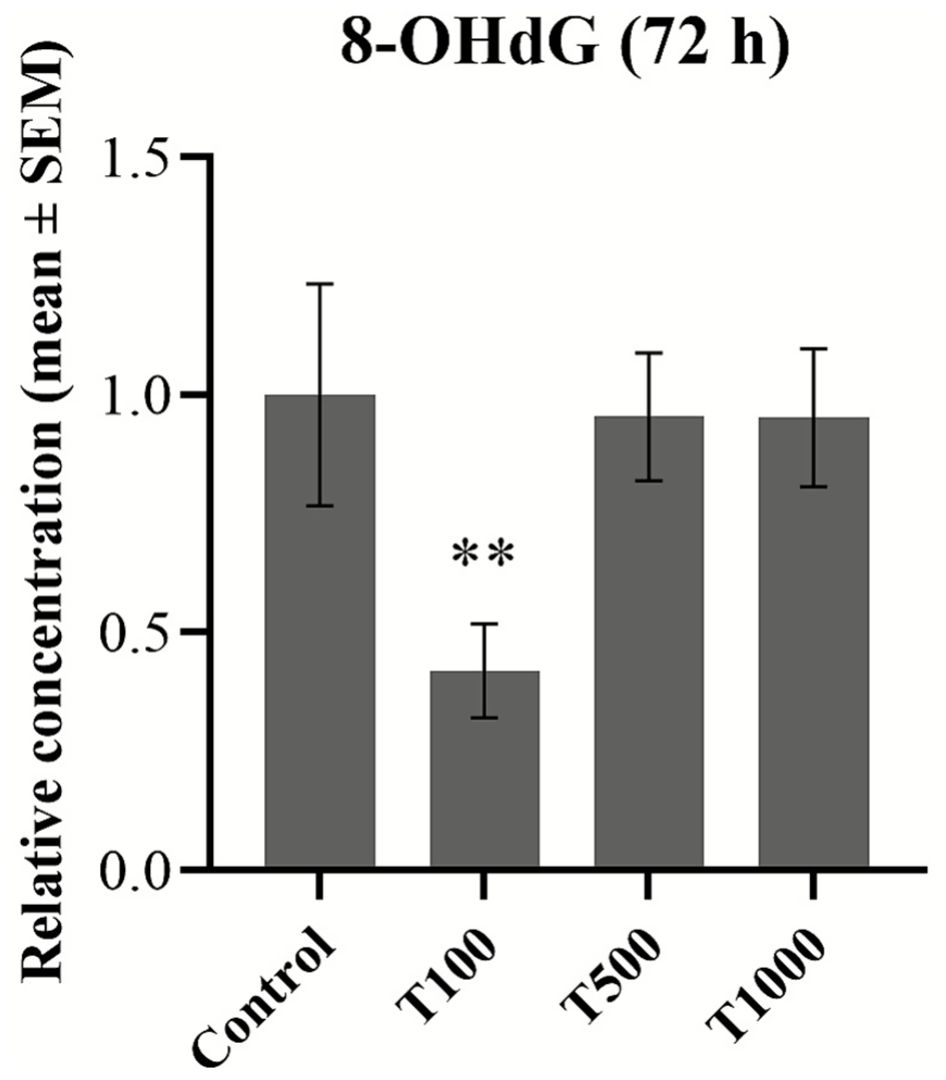
Effects 72 h T-2 toxin treatments on the 8-hydroxy-2′-deoxyguanosine (8-OHdG) content of primary hepatic 3D cell co-cultures of chicken origin, assessed by chicken-specific ELISA tests from the cell lysates. Control: cells without T-2 toxin exposure; T100: 100 nM, T500: 500 nM, T1000: 1000 nM T-2 toxin treatment. Relative concentrations were calculated by considering the mean value of the control group as 1. Results are expressed as mean ± SEM. Significant difference from the control group is indicated by *. * *p* < 0.05.

In order to assess the potential oxidative stress, the extracellular concentrations of MDA and HSP27, as well as the intracellular amount of 8-OHdG were measured. Following 48 h treatment, both the MDA and the HSP27 production of the cells was significantly decreased (*p* = 0.006; *p* < 0.010; *p* = 0.033 in the case of MDA; while *p* = 0.017; *p* = 0.017; *p* = 0.002 in the case of HSP27, respectively) (Figures 2a, 3a). No significant changes were detected after the 72 h treatment (Figures 2b, 3b). The 8-OHdG content of the cells was significantly reduced (*p* = 0.009) by the lowest T-2 toxin concentration after 72 h of treatment (Figure 4). The mean ± SEM values obtained from the MDA, HSP27 and 8-OHdG measurements and the corresponding significances are shown in Supplementary Table 1.

### 3.3 Inflammation

**Figure 5:**
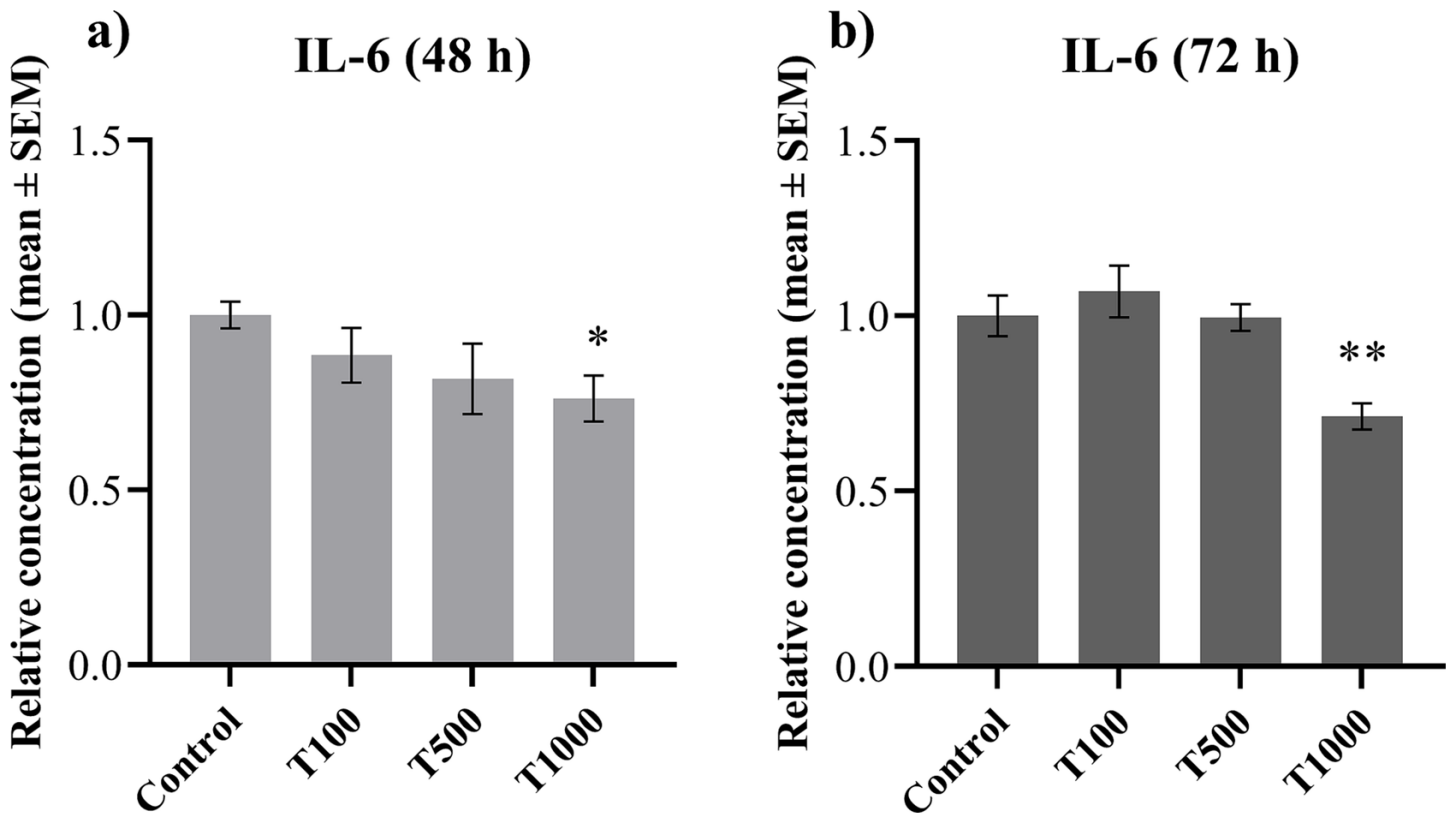
Effects of 48 and 72 h T-2 toxin treatments on the interleukin-6 (IL-6) content of primary hepatic 3D cell co-cultures of chicken origin, assessed by chicken-specific ELISA tests. Concentration of IL-6 after **(a)** 48 h and **(b)** 72 h of treatment. Control: cells without T-2 toxin exposure; T100: 100 nM, T500: 500 nM, T1000: 1000 nM T-2 toxin treatment. Relative concentrations were calculated by considering the mean value of the control group as 1. Results are expressed as mean ± SEM. Significant difference from the Control group is indicated by *. * *p* < 0.05; **p < 0.01.

**Figure 6:**
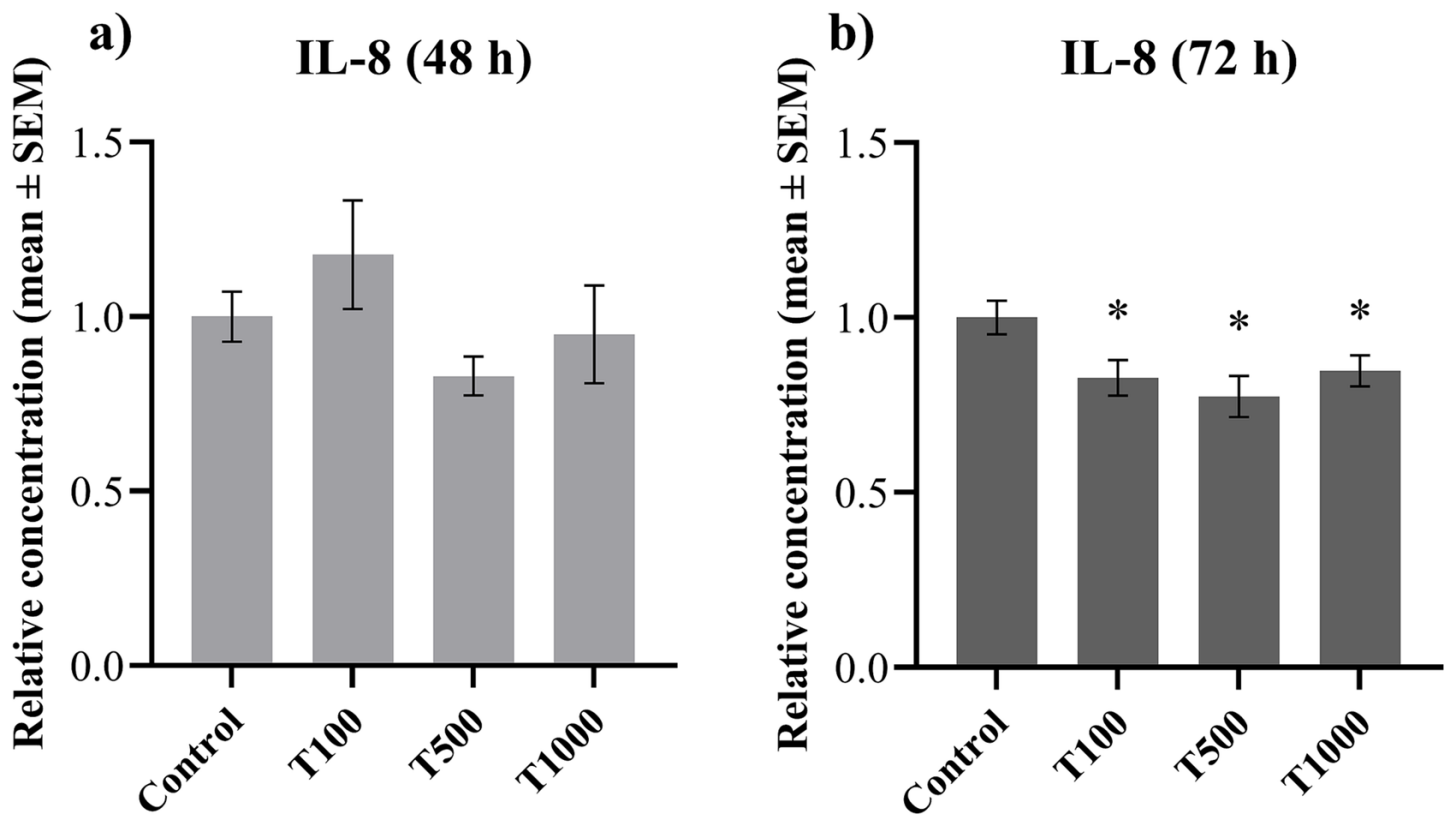
Effects of 48 and 72 h T-2 toxin treatments on the interleukin-8 (IL-8) content of primary hepatic 3D cell co-cultures of chicken origin, assessed by chicken-specific ELISA tests. Concentration of IL-8 after **(a)** 48 h and **(b)** 72 h of treatment. Control: cells without T-2 toxin exposure; T100: 100 nM, T500: 500 nM, T1000: 1000 nM T-2 toxin treatment. Relative concentrations were calculated by considering the mean value of the control group as 1. Results are expressed as mean ± SEM. Significant difference from the control group is indicated by *. * *p* < 0.05.

To investigate the inflammatory processes, the extracellular concentrations of IL-6 and IL-8 were determined. The concentration of IL-6 was significantly lowered (*p* = 0.010, *p* = 0.002, respectively) by the 1,000 nM T-2 toxin treatment both after 48 and 72 h (Figures 5a,b). The IL-8 concentration was significantly decreased (*p* = 0.033; *p* = 0.014; *p* = 0.042, respectively) after 72 h in every treatment group (Figure 6b), but no significant changes were observed after 48 h of toxin treatment (Figure 6a). The mean ± SEM values obtained from the IL-6 and IL-8 measurements and the corresponding significances are shown in Supplementary Table 1.

## 4 Discussion

In this study, primary 3D cell cultures of chicken origin were used to investigate the effects of T-2 toxin. In these models, magnetic nanoparticles are added to the cell cultures to magnetize the cells, causing them to aggregate into spheroids at the bottom of a multiwell plate when exposed to cylindrical magnets (37). Several preliminary studies have shown that these nanoparticles do not affect cell growth and physiology (36, 40). The concentrations of T-2 toxin used were based on preliminary literature data of *in vitro* studies and prior experiments by our research group (8, 9). Although direct extrapolation of *in vitro* concentrations to feed contamination levels is challenging due to differences in the animal’s absorption, metabolism and distribution of the toxin, the lower concentrations are considered representative for subclinical exposure levels that could occur in feed under moderate *Fusarium* contamination (9, 41, 42). The average amount of T-2 toxin in feed can vary between 20 and 50 μg/kg, which corresponds to a very low daily intake (41, 43). Hence, the applied 100 nM (46.65 ng/mL) T-2 toxin level corresponds to the tissue concentrations possibly occurring *in vivo* following subclinical toxin exposure. The applied higher concentrations represent a more severe exposure, providing a clear cellular challenge to study dose-dependent effects *in vitro*. While this may represent a limitation of *in vitro* studies and should certainly be taken into consideration in future *in vivo* studies, it also enables a more precise characterization of toxin-induced cellular processes, as a wide range of concentrations can be investigated.

T-2 toxin binds to various proteins, thus inhibiting the activity of several key enzymes essential for the cellular catabolic processes, such as succinate dehydrogenase or mitochondrial NADH dehydrogenase (9, 44, 45). Such interference can lead to cellular energy deficiency. Therefore, the metabolic activity of the cells was investigated by CCK-8 assay, which also indicates the overall viability of the cells. The results show that T-2 toxin treatment led to a moderate decrease in the metabolic activity of the cells after both 48 and 72 h. However, as the average decline remained below 25%, the observed effect cannot be considered cytotoxic. These findings are in line with our previous reports describing the viability-decreasing effect of the T-2 toxin both in 2D and 3D hepatic cell cultures of chicken origin (8, 9). Importantly, the cell cultures were successfully maintained for 72 h, with preserved viability, as reflected by the absence of significant differences between the means of the control groups. Comparing the results with our previous study on the effect of T-2 toxin after 24 h, it is clear that after 24 and 48 h, all toxin concentrations reduced the cellular viability, but after 72 h, only the highest toxin concentration was able to decrease the metabolic activity (9). This suggests a possible metabolic adaptation in the liver cells, as the metabolic depression was alleviated after the longer incubation period, suggesting a partial recovery after longer toxin exposure. During the adaptive response, cells promote homeostasis through the regulation of various cellular and extracellular functions. This type of response to exogenous stressors is beneficial for the cells – and for the organism as a whole – because it can improve the response capacity to chemically induced stress, is reversible, and preserves viability (46).

T-2 toxin is an amphipathic molecule capable of entering the cellular lipid bilayer membranes, where it stimulates lipid peroxidation and MDA generation by increasing ROS production (17). In contrast, our results show that the amount of MDA decreased after 48 h of treatment. Similarly, the levels of HSP27, a molecular chaperone whose production is increased in response to various stressors, were also reduced after 48 h (47, 48). HSP27 interacts with the oxidized or misfolded proteins and forms transient structures with them, thereby limiting protein aggregation (25). These findings are also consistent with our previous observations following 24 h of toxin exposure, where a similar fluctuation in both oxidative parameters was detected (9). In this experiment, only the 1,000 nM toxin exposure could reduce the MDA level, while there was no significant change in the amount of HSP27. On the other hand, after 48 h, all three toxin concentrations reduced their levels, and after 72 h, there were no significant changes observed. These results suggest that the oxidative damage of phospholipids along with the provoked compensatory mechanisms may require a longer time to be initiated as reflected by the toxin’s more pronounced action at 48 h. The concurrent decrease in both MDA and HSP27 levels likely reflects the activation of multiple cellular processes to compensate the adverse effects of the toxin (8, 9). T-2 toxin influences cellular oxidative homeostasis, amongst others by stimulating or reducing the expression of the transcription factor nuclear factor erythroid 2-related factor 2 (Nrf2). Upon Nrf2 up-regulation, the transcriptional factor induces the production of antioxidant enzymes, which helps to restore the normal homeostasis in the cells (5, 9, 49). This is reflected in the corresponding decrease in the HSP27 concentration, which indicates that the rate of HSP27 consumption exceeded the rate of production in order to restore the physiological conditions in the cells (38). Nonetheless, by the end of the 72 h incubation, this balance appeared to be re-established, as reflected by stable MDA levels and the normalization of metabolic activity. Thus, the adaptation of the liver cells mentioned above is further supported.

The levels of 8-OHdG were only measured after 72 h of treatment from the cell lysates, and they showed a significant decrease with 100 nM T-2 toxin treatment. This reduction could also suggest the induction of protective mechanisms in the cells. Notably, changes in the 8-OHdG levels did not show a dose-dependent relationship with the toxin treatment. This phenomenon may be explained by the so-called non-monotonic dose–response (NMDR), which has been described for several compounds. The fundamental hypothesis is that classical dose responses (DR) show a dose-dependent monotonic trend (50). In contrast, in the case of NMDR, the DR curve shifts its slope in response to a specific dose (51). This pattern has been primarily observed for hormones and pharmaceuticals, although other compounds could also exhibit such DR. However, the precise conditions under which this response occurs remain unclear (50, 52).

The cellular response to T-2 toxin treatment showed a similar trend in the IL-6 levels after both incubation periods. IL-6 concentrations were reduced both after 48 and 72 h by the highest toxin treatment. Interestingly, the lowest toxin concentration was able to significantly lower the amount of IL-6 after 24 h of treatment in our previous study (9). These results further support the toxin-associated immunosuppression and suggest the involvement of autophagy, a cellular process responsible for the removal of irregular, dysfunctional components (9, 35). In addition, autophagy has been shown to limit inflammatory cytokine release from macrophages during fibrogenesis (53). T-2 toxin may regulate this process through autophagy-related gene 5 (ATG5) and mechanistic target of rapamycin (mTOR) regulation (9, 54). However, following prolonged exposure, it is likely that the cells adapted to some extent to the toxin treatment, necessitating the highest applied toxin concentration to elicit reductions the IL-6 levels.

The concentration of IL-8 showed a significant decrease only after 72 h of incubation with all three treatment concentrations. In contrast, after 24 h the lowest toxin concentration increased the amount of IL-8, while no significant changes were observed after 48 h exposure (9). This may suggest that the potential immunostimulatory activity of T-2 toxin has been attenuated after shorter exposure. However, the opposite effect was observed after 72 h, which may indicate an immunosuppressive action of the toxin, being in line with the previously described dose-dependent pro-inflammatory cytokine-decreasing effect of several mycotoxins (55). Another possible explanation for the decline in the IL-8 levels can be the abovementioned mechanism of autophagy (9, 35, 53).

In comparison with our previous results after 24 h of T-2 toxin treatment, it was found that the prolonged toxin treatment induced defence mechanisms in the cells, which then resulted in effective cellular adaptation concerning the metabolic activity and redox homeostasis. During the inflammatory response, the stimulation of IL-8 release after 24 h has turned to immunosuppression following 72 h exposure, indicating the time-dependent dynamics of the toxin-provoked inflammatory response. In conclusion, it became clear that T-2 toxin exerted a dose- and time-dependent effect on the liver cells. Moreover, the successful maintenance of the cell cultures for up to 72 h supports the suitability of this model for investigating longer-term treatments.

Although this study details the cellular inflammatory and oxidative responses to T-2 toxin, these results may also be useful in understanding systemic processes and may have practical implications. *In vivo* experiments showed that dietary exposure to different concentrations of T-2 toxin impaired feed intake and feed conversion ratio, decreased body weight, and reduced protein, cholesterol and hemoglobin levels in the serum (56). In addition, exposure to T-2 toxin can diminish responsiveness to vaccines and increases the animal’s susceptibility to infections that cause economic damage worldwide (12–14). All of these growth performance impairments are mostly related to the metabolic, immunological and oxidative damage examined in this study (9, 56). It can be concluded from these results that even subclinical cellular stress and immunosuppression can negatively affect flock-level productivity and reduce growth performance, highlighting the importance of continuous monitoring and elimination of T-2 toxin contamination in poultry feed.

## 5 Conclusion

These results confirmed that the magnetic 3D hepatic cell *co-cultures of chicken origin* used in this experiment can be maintained for longer periods of time than traditional monolayer cell cultures, allowing longer exposures to be tested. The used concentrations of T-2 toxin, while having adverse effects on the cells, were not found to be cytotoxic. The toxin affected cellular oxidative homeostasis by inducing cellular compensatory mechanisms to protect the cells against its harmful effects. In addition, it had a dose- and time-dependent effect on the cellular immunity and possibly induced autophagy in the cells. However, hepatocytes were supposedly able to adapt to prolonged toxin exposure as seen in the cellular metabolic activity and oxidative parameters. Our results underline the significance of routine mycotoxin monitoring and elimination methods since these data suggest that subclinical T-2 toxin exposure may affect liver metabolism and the immune system in chickens.

## Data Availability

The raw data supporting the conclusions of this article will be made available by the authors, without undue reservation.
