# Supplementary material for: Role of T-2 toxin in the modulation of oxidative homeostasis and immune function in three-dimensional hepatic cell cultures of chicken origin
Source: Front Vet Sci. 2026 Feb 10;13:1759841. doi: 10.3389/fvets.2026.1759841 (PMC12929105; doi:10.3389/fvets.2026.1759841)
Supplement: Supplementary file 1 [file Supplementary_file_1.docx]

***Supplementary Material***

# **Supplementary Table 1.**

Means of the different measurements with the corresponding SEM. Control: cells without T-2 toxin exposure; T100: 100 nM, T500: 500 nM, T1000: 1000 nM T-2 toxin treatment. Significant difference from the Control group is indicated by *. * p < 0.05; ** p < 0.01; *** p < 0.001.
